# Investigating aluminum cookpots as a source of lead exposure in Afghan refugee children resettled in the United States

**DOI:** 10.1038/s41370-022-00431-y

**Published:** 2022-05-02

**Authors:** Katie M. Fellows, Shar Samy, Yoni Rodriguez, Stephen G. Whittaker

**Affiliations:** 1 Hazardous Waste Management Program in King County, Seattle, WA USA; 2 Department of Environmental and Occupational Health Sciences, University of Washington, Seattle, WA USA

## Abstract

**Background:**

Afghan refugee children resettled in Washington State have the highest prevalence of elevated blood lead levels (BLLs) of any other refugee or immigrant population. Resettled families brought several lead-containing items with them from Afghanistan, including aluminum cookpots.

**Objectives:**

To evaluate the potential contribution of lead-containing cookpots to elevated BLLs in Afghan children and determine whether safer alternative cookware is available.

**Methods:**

We screened 40 aluminum cookpots for lead content using an X-ray fluorescence (XRF) analyzer and used a leachate method to estimate the amount of lead that migrates into food. We also tested five stainless steel cookpots to determine whether they would be safer alternatives.

**Results:**

Many aluminum cookpots contained lead in excess of 100 parts per million (ppm), with a highest detected concentration of 66,374 ppm. Many also leached sufficient lead under simulated cooking and storage conditions to exceed recommended dietary limits. One pressure cooker leached sufficient lead to exceed the childhood limit by 650-fold. In contrast, stainless steel cookpots leached much lower levels of lead.

**Significance:**

Aluminum cookpots used by refugee families are likely associated with elevated BLLs in local Afghan children. However, this investigation revealed that other U.S. residents, including adults and children, are also at risk of poisoning by lead and other toxic metals from some imported aluminum cookpots.

**Impact Statement:**

Some aluminum cookware brought from Afghanistan by resettled families as well as cookpots available for purchase in the United States represent a previously unrecognized source of lead exposure.

## Introduction

### Childhood lead poisoning—a worldwide public health problem

Childhood lead poisoning is one of the most preventable noninfectious diseases, and yet it remains one of the most common pediatric health problems worldwide. Children are at risk for serious health effects from exposure to this metal that has no physiological function and is toxic at any level of exposure [1]. The literature describing the health effects of lead is extensive and lead toxicity has been described in every organ system that has been rigorously studied. Children are particularly susceptible to adverse health outcomes from lead exposures. Of special concern is that low-level exposures can affect a child’s neurological development, with severe impacts on learning, intelligence, and behavior [2]. Cardiovascular disease has also been associated with low-level exposures to lead and cadmium [3].

Previously identified major sources of children’s exposure to lead include: leaded gasoline, active industries (e.g., mining), paints and pigments, solder in food cans, ceramic glazes, drinking-water systems with lead solder and lead pipes, products (such as herbal and traditional medicines, folk remedies, cosmetics, and toys), incineration of lead-containing waste, electronic waste, the food chain (via contaminated soil), and lead contamination from former industrial sites [4]. Refugees from countries where these exposures are endemic bring a body burden of lead with them when they arrive in the United States (U.S.). These children may then be further exposed to lead once they have been resettled—from items their families brought with them from their countries of origin and other sources within the U.S.

### Lead health surveillance in the United States

The Centers for Disease Control and Prevention (CDC) recommends actions for follow-up and case management for children with blood lead levels (BLLs) at or above its blood lead reference value (BLRV), with escalating levels of intervention with increasing BLL [5]. In November 2021, the CDC lowered the BLRV for children it established in 2012 from 5 micrograms per deciliter of whole blood (μg/dL) to 3.5 μg/dL [6]. However, it is important to note that the BLRV is not a health-based value. Rather, it is a screening tool to identify children who have higher BLLs compared with most children [6].

In 2021, Washington State, Snohomish County Public Health, and Public Health-Seattle & King County (PHSKC) received funding through the CDC’s National Childhood Lead Poisoning Prevention Program [7]. Elevated BLLs are notifiable conditions in Washington State. Consequently, all blood lead test results are reportable to the Washington State Department of Health (WA DOH) [8]. The blood lead results are then transmitted to local health jurisdictions for follow-up in their communities based on the child’s residence address. In King County, Washington, case investigations for children with BLLs at or above 5 μg/dL are led by investigators from the Hazardous Waste Management Program (Haz Waste Program), based in Seattle, Washington.

### Afghan refugee children—a population at risk

Several studies have demonstrated that lead poisoning disproportionately impacts refugee children resettled in the United States and they are susceptible to further exposures in the U.S. due to substandard housing [9, 10]. Consequently, the CDC recommends that all refugee infants, children, adolescents, and pregnant and lactating women and girls arriving in the U.S. undergo an initial lead exposure screening with a blood lead test [11].

The WA DOH’s Refugee and Immigrant Health Program reported that for Federal Fiscal Year 2016–2020, children from Afghanistan (0–16 years of age) who resettled in Washington State (BLL data available for 1669 Afghan children) had the highest prevalence of BLLs of 5–9 μg/dL (34%) and ≥10 μg/dL (10%) of all resettled children [12].

Elevated BLLs in Afghan children were also noted in a multi-state study of Special Immigrant Visa (SIV) holders from Iraq and Afghanistan after arrival in the U.S. In 2014–2016, 47.8% of children from Afghanistan had BLLs of 5–9 μg/dL; 10.9% had BLLs of 10–19 μg/dL [13]. In an earlier study of resettled refugees who arrived in the U.S. between 2010 and 2014, children from Afghanistan had the highest prevalence of BLLs ≥ 10 μg/dL (16.7%) of children from any other country of origin [14].

### Interventions in King County, Washington

Recognizing the high prevalence of elevated BLLs in newly resettled Afghan refugee children, the Haz Waste Program and partners from PHSKC conducted a focused intervention between July 2018 and February 2020 (the “Public Health Partnership”). This intervention included in-home environmental assessments at the primary residences of Afghan children with BLLs ≥5 μg/dL. The environmental assessment included screening items for lead content using an X-ray fluorescence (XRF) analyzer, which revealed that aluminum cookpots brought by the families from Afghanistan (including traditional Afghan pressure cookers and cookware pots) frequently contained lead levels in the hundreds of parts per million (ppm) and occasionally in the thousands of ppm (unpublished data).

### Cookpots as a source of lead exposure

Most studies of lead-contaminated dinnerware and cookware have focused primarily on lead-glazed ceramics used for cooking and storing food [15–27]. Leaching of lead typically increases with use of the dish, as the glaze deteriorates through heat and mechanical energy (i.e., grinding and cooking) [23, 25]. The tendency of lead to leach from ceramicware is also influenced by glaze composition, food pH, temperature, physical state of the food, and duration of food contact [16–18, 20, 21].

Aluminum cookware is popular with consumers because of its low price, light weight, and efficient heat conduction. However, aluminum cookpots are significant sources of lead exposure, especially in the developing world [28–37]. Artisanal aluminum cookware associated with lead exposures is typically locally-made, uncoated, non-anodized, and made from discarded scrap metal. The sources of scrap metal vary, but investigations in Cameroon and other West African countries found that the smelting process often used drinking cans, car and motorbike engine parts, vehicle radiators, transmissions, airplane fuselages, lead batteries, computer and electronic components, and other materials [28, 32, 35, 36]. Several examples of manufacturing aluminum cookpots from scrap materials are depicted on the online video platform, YouTube (https://www.youtube.com/results?search_query=making+aluminum+pan). This practice likely occurs throughout the developing world [37, 38], occasionally resulting in lead poisoning in surrounding communities [39, 40].

As with ceramic cookware, previous studies found that leachability from aluminum increases with temperature. Heating cookware for 2 h or more leached considerably more lead into food compared to storing food at ambient temperature for 24 h or more [28, 35, 41]. The age and amount of use of the aluminum cookware can also affect the migration of metals into food. Products tested by repeated boiling and cooking operations suggest that leaching of lead and other toxic metals, such as cadmium, may increase with use—several cooking pots released considerably more lead on subsequent extractions or in older, used pots [28, 32, 34]. Acidic foods and solutions also increase lead extraction, compared to neutral or more basic substances [31, 33, 42].

### Stainless steel as a safer alternative

In a study of trace elements found in foods from Sub-Saharan Africa, aluminum and lead concentrations were significantly lower when traditional foods were prepared in stainless steel cookware, compared to aluminum. The authors concluded that dietary exposures to toxic metals would be reduced by using stainless steel [31].

The American Iron and Steel Institute (AISI) defines alloy steels as iron-based mixtures where manganese is greater than 1.65%, silicon is over 0.5%, copper is above 0.6%, or other minimum quantities of alloying elements such as chromium, nickel, molybdenum, or tungsten are present. AISI defines “stainless steel” as grades of steel that contain more than 10% chromium, with or without other alloying elements [43]. These stainless steels achieve their stainless characteristics through the formation of an invisible and adherent chromium-rich oxide film [44]. Lead is not typically a component of stainless steel, other than for specific applications that require enhanced machinability [45].

### Present study

Previous studies suggest that any critical evaluation of lead exposures from cookware should simulate both the cooking times and typical uses. Consequently, in addition to conducting XRF analysis for lead content, we developed a novel leachate method to estimate the lead dose from consuming food in these cookpots under simulated cooking and storage conditions. We included stainless steel items to determine whether they represent safer alternatives to aluminum.

Therefore, the goals of this present study were to: (1) measure the lead content of cookpots used by the Afghan refugee community, (2) measure the amount of lead that leaches from the cookpots under simulated cooking and storage conditions, and (3) determine whether stainless steel represents a safer alternative.

## Methods

### Acquisition of cookpots

We acquired 45 cookpots for analysis between November 2019 and May 2021. These cookpots originated from several sources, including donations of used aluminum pots, pans, and traditional pressure cookers from Afghan families. We also purchased similar aluminum cookpots from retail outlets, including Amazon, Etsy, and AliBaba. Finally, to identify a potentially safer alternative for community use, we purchased new/unused stainless steel cookpots for analysis.

### XRF analysis of cookpots

Lead screening was conducted between November 2019 and June 2021 using a handheld Bruker S1 Titan XRF analyzer (Bruker Corporation, Billerica, Massachusetts). More details are provided in the Supplemental Information, including determining the XRF analyzer’s response to a series of lead standards in an aluminum alloy matrix (see Table S-1 and Fig. S-1).

Although the focus of this investigation was lead, data were also recorded for other routinely detected metals, including Al, Cr, Mn, Fe, Co, Ni, Cu, As, and Cd. Data for these additional metals were reviewed when determining the composition of individual cookpot components and evaluating metals migrating from stainless steel.

**Fig. 1:**
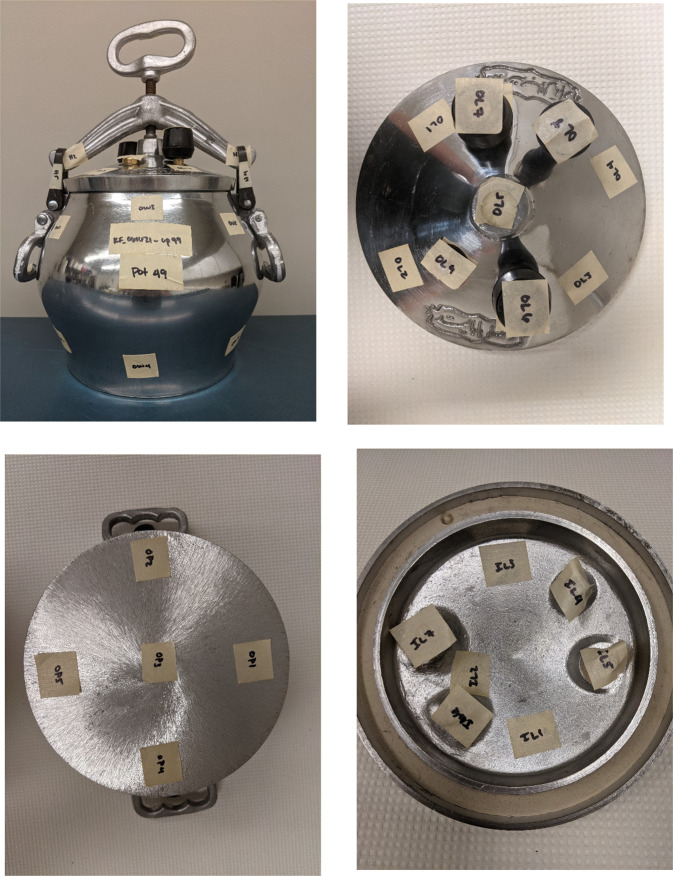
Example XRF testing locations on an aluminum Afghan pressure cooker (Pot #49). Top left: Labeled pressure cooker body. Bottom left: Labeled base. Top right: Labeled outer surface of lid. Bottom right: Labeled inner surface of lid.

**Fig. 2:**
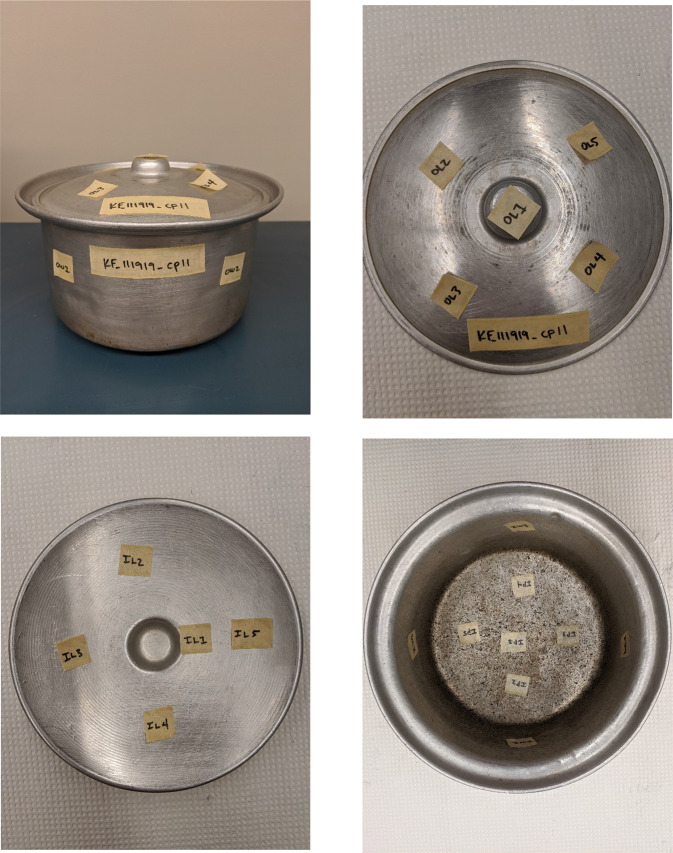
Example XRF testing locations on a typical aluminum cookpot (Pot #11). Upper left: Labeled body of cookpot. Lower left: Labeled inner surface of lid. Upper right: Labeled outer surface of lid. Bottom right: Labeled inner surface of cookpot.

Each cookpot was screened using the XRF analyzer at between 20 and 50 locations. Screening was typically conducted once in each quadrant and in the center of large sections of the cookpot. Smaller sections, such as handles, inserts, and rivets, were typically tested at one location. If the pot was too small to accommodate the XRF analyzer, only the lip or rim of the inner surface was screened. The sampling strategy is summarized in Table 1. Photographs of representative cookpots, with removable labels applied at the sampled locations, are provided in Figs. 1 and 2. Labeling these locations allowed us to verify measurements, when necessary.

**Table 1:** Typical XRF screening of cookware.

Cookware section	Number of tested locations^a^
Lid handle	1
Outer surface of lid	4
Inner surface of lid (including handle attachments)	5
Outer wall of cookpot	4
Inner wall of cookpot	4
Inner surface of cookpot base	5
Outer surface of cookpot base	5

^a^The number of XRF locations tested varied according to the size of the cookware and the presence of a lid, handle, inserts, and other hardware.

XRF results were provided in ppm with an error term that represents three times the standard deviation (i.e., the 3-sigma limit). At the end of the sampling session, data were transferred from the instrument using Bruker Instrument Tools (BIT) software and stored/further processed in Microsoft Excel^TM^.

Data for cookpots with more than 50% of measurements below the limit of detection (LOD) should be considered tentative and are highlighted in Tables 2 and 3. This issue is discussed further in the Supplemental Information.

**Table 2:** XRF and leachate results for aluminum cookware.

Pot #	Description	Country of Origin	Purchased or Donated	Median Pb Level (XRF, ppm)	Range of Pb Levels (XRF, ppm)	Lead leachate conc. at 15 min (μg/mL)	Lead dose/serving at 15 min (μg/250 mL)	Lead leachate conc. at 24 h (μg/mL)	Lead dose/serving at 24 h (μg/250 mL)
1	Caldero	Unknown	Purchased	10^b^	(0, 19)	0.00065	0.163	0.00657	1.64
2	Caldero	Colombia	Purchased	96^b^	(0, 225)	0.0272	6.80^d^	0.21	52.5^e^
3	Caldero	Unknown	Purchased	12	(0, 26)	0.00074	0.185	0.00983	2.46
4	Cookpot	Unknown	Purchased	10^b^	(0, 192)	0.00026	0.065	0.0031	0.775
5	Multi-level Steamer	Taiwan	Purchased	24	(10, 47)	0.214	53.5^e^	0.226	56.5^e^
6	Steamer	China	Purchased	28	(0, 44)	0.0008	0.200	0.0138	3.45^d^
7	Cookpot	China	Purchased	8^b^	(0, 18)	0.00006	0.015	0.00029	0.0725
8	Multi-level Steamer	Unknown	Purchased	10^b^	(0, 85)	0.0009	0.225	0.0113	2.83
9	Pressure Cooker	Afghanistan^a^	Donated	222	(0, 68,926)^c^	0.0422	10.6^d^	0.0957	23.9^e^
10	Cookpot	Afghanistan^a^	Donated	14,318	(7148, 17,094)	1.59	397^e^	4.06	1015^e^
11	Cookpot	Afghanistan^a^	Donated	2807	(803, 4182)	0.0808	20.2^e^	0.14	35.0^e^
12	Cookpot	Afghanistan^a^	Donated	8807	(2751, 18,599)	0.359	89.8^e^	1.26	315^e^
13	Cookpot	Afghanistan^a^	Donated	634	(217, 742)	0.0152	3.80^d^	0.0276	6.90^d^
14	Cookpot	Afghanistan^a^	Donated	360	(157, 714)	0.154	38.5^e^	0.274	68.5^e^
15	Caldero	China	Purchased	10^b^	(0, 367)	0.00041	0.103	0.00599	1.50
16	Caldero	Colombia	Purchased	138	(0, 183)	0.00952	2.38	0.249	62.3^e^
17	Skillet	Unknown	Purchased	387	(8, 489)	0.0149	3.73^d^	0.424	106^e^
18	Stock Pot	China	Purchased	0^b^	(0, 19)	0.0004	0.100	0.0013	0.325
22	Pressure Cooker	India	Purchased	8^b^	(0, 3828)^c^	0.00007	0.0175	0.00191	0.478
29	Pressure Cooker	Unknown	Purchased	595	(103, 53,668)^c^	0.0141	3.53^d^	2.12	530^e^
30	Cookpot	China	Purchased	28	(10, 260)	0.00127	0.318	0.00651	1.628
31	Cookpot	India	Purchased	95	(0, 374)	0.0072	1.80	0.0632	15.8^e^
32	Cookpot	Unknown	Purchased	476	(12, 554)	0.0155	3.83^d^	0.138	34.5^e^
33	Cookpot	Unknown	Purchased	11^b^	(0, 203)	0.00053	0.133	0.00278	0.695
34	Cookpot	Unknown	Purchased	0^b^	(0, 134)	0.0002	0.050	0.0002	0.050
35	Caldero	Unknown	Purchased	11^b^	(0, 124)	0.0007	0.175	0.00395	0.988
36	Pressure Cooker	Unknown	Purchased	42	(0, 78)^c^	0.00276	0.690	0.0287	7.18^d^
37	Cookpot	Afghanistan^a^	Donated	4,660	(0, 33,062)	0.154	38.5^e^	0.417	104^e^
38	Pressure Cooker	Unknown	Purchased	12^b^	(0, 5,323)^c^	0.00099	0.248	0.00328	0.820
39	Cookpot	Afghanistan^a^	Donated	2016	(1,469, 56,950)	0.117	29.3^e^	0.37	92.5^e^
40	Cookpot	Afghanistan^a^	Donated	3553	(2582, 29,429)	0.191	47.8^e^	0.56	140^e^
41	Cookpot	Afghanistan^a^	Donated	1533	(0, 43,643)	0.0248	6.20^d^	0.0342	8.55^d^
42	Cookpot	Afghanistan^a^	Donated	301	(0, 440)	0.118	29.5^e^	0.221	55.3^e^
43	Cookpot	Afghanistan^a^	Donated	3117	(662, 28,287)	0.0499	12.5^e^	0.189	47.3^e^
44	Cookpot	Afghanistan^a^	Donated	4546	(3162, 32,612)	0.0877	21.9^e^	0.132	33.0^e^
45	Pressure Cooker	Afghanistan^a^	Donated	5063	(0, 40,158)^c^	0.258	64.5^e^	0.584	146^e^
46	Pressure Cooker	Afghanistan^a^	Donated	393	(0, 43,900)^c^	0.067	16.8^e^	0.104	26.0^e^
47	Pressure Cooker	Afghanistan^a^	Purchased	556	(8, 37,040)^c^	0.195	48.8^e^	6.3	1580^e^
48	Pressure Cooker	Afghanistan^a^	Purchased	693	(0, 66,374)^c^	0.284	71.0^e^	7.77	1940^e^
49	Pressure Cooker	Afghanistan^a^	Purchased	497	(0, 53,425)^c^	0.279	69.8^e^	7.11	1780^e^

^a^Assumed to be from Afghanistan, but the country of origin is unclear because several countries (i.e., China, India, and Pakistan) manufacture cookware for sale in Afghanistan.

^b^More than 50% of XRF measurements <LOD.

^c^Highest lead concentration detected in the pressure cooker vent pipe.

^d^Estimated dose ≥ child IRL (3 µg/day) and < adult IRL (12.5 µg/day).

^e^Estimated dose ≥ adult IRL (12.5 µg/day).

**Table 3:** XRF and leachate results for stainless steel cookware.

Pot #	Description	Country of Origin	Purchased or Donated	Median Pb Level (XRF, ppm)	Range of Pb Levels (XRF, ppm)	Lead leachate conc. at 15 mins (μg/mL)	Lead dose/serving at 15 mins (μg/250 mL)	Lead leachate conc. at 24 h (μg/mL)	Lead dose/serving at 24 h (μg/250 mL)
19	Stock Pot	China	Purchased	16^a^	(0, 84)	0.0002	0.050	0.0002	0.050
20	Pressure Cooker	Unknown	Purchased	20^a^	(0, 77)^b^	0.00033	0.0825	0.00033	0.0825
21	Pressure Cooker	China	Purchased	13^a^	(0, 104)^b^	0.0015	0.375	0.00006	0.015
23	Pressure Cooker	China	Purchased	17^a^	(0, 86)	0.00018	0.045	0.0002	0.050
28	Pressure Cooker	Spain	Purchased	15^a^	(0, 132)^b^	0.00058	0.145	0.00054	0.135

^a^More than 50% of XRF measurements <LOD.

^b^Highest lead concentration detected in the pressure cooker vent pipe.

### Leachate analysis of cookpots

There are no standard methods for the measurement of migration of lead or other metals from metal cookware, nor do any standard methods mimic typical cooking or food storage. Therefore, we modified the standard procedures described in ASTM International’s Standard Test Method C 738-94 [46] and the U.S. Food & Drug Administration’s (FDA’s) Elemental Analysis Manual (EAM) Method 4.1 [47].

We compared our results to the FDA’s interim reference levels (IRLs) for lead, which are the maximum daily dietary intakes of lead from food. The FDA derived an IRL of 3 µg/day for children and 12.5 µg/day for women of childbearing age to maintain BLLs below CDC’s previous BLRV of 5 µg/dL [48]. Serving size was assumed to be 250 mL, or approximately one cup, based on a previous study [35] and the FDA’s guidance that one cup of a mixed dish (e.g., a casserole or stew) is consumed per meal [49].

Prior to experimentation, cookpots were washed with tap water and mild dish soap, triple rinsed with ultra-pure deionized (DI) water (18.2 Mohm) and allowed to dry. Vessels were filled with diluted acetic acid (Glacial, Fisher TraceMetal^TM^ Grade) in DI water (4% v/v), then heated to a simmer using a cast iron electric burner (Cuisinart Model CB-60P1) at the highest temperature setting. After the liquid had simmered for 15 min, the cookpot was removed from the heat and a 100 mL aliquot of liquid was taken for testing. The cookpot was then allowed to sit for an additional 24 h at 20–24 °C (68–75 °F), when a second 100 mL aliquot was taken. Samples were stored at 4 °C before being transported to the University of Washington’s Environmental Health Laboratory (Seattle, WA) for preservation. Analysis was conducted via Inductively Coupled Plasma-Mass Spectrometry (ICP-MS), based on EPA methods [50, 51] that were optimized for metals determination in acetic acid solution [52] (see the Supplemental Information for additional details).

## Results

### Aluminum cookpots

We conducted XRF analyses and leachate testing on 40 aluminum cookpots. Of these, 15 were previously used and donated by Afghan community members. We also purchased 25 unused aluminum cookpots.

**Fig. 3:**
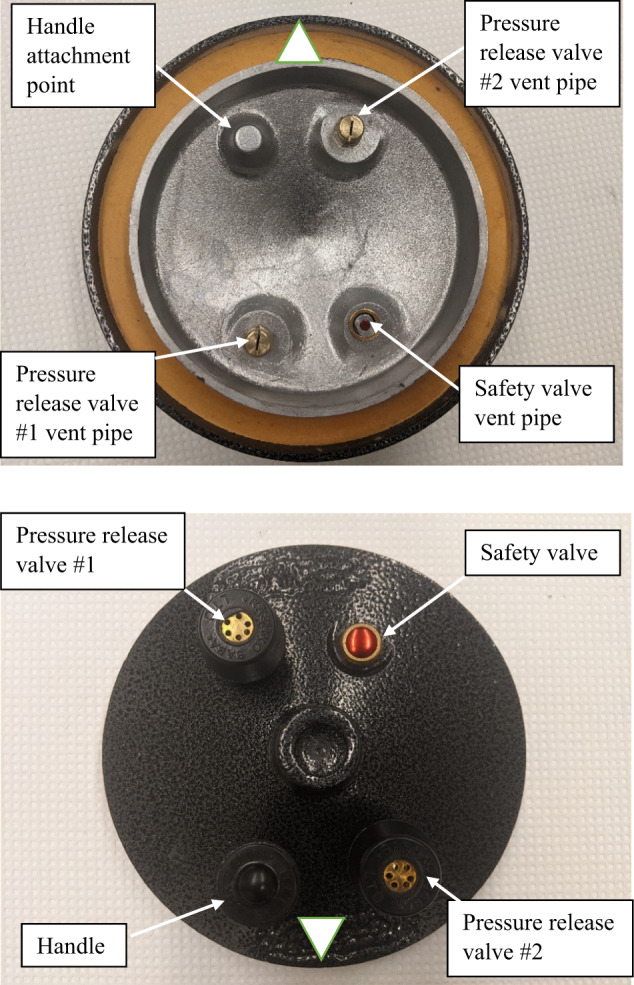
Lid of a traditional Afghan pressure cooker (Pot #48). Top: Inner surface of pressure cooker lid. Bottom: Outer surface of pressure cooker lid.

The results of the XRF screening and leachate analyses for aluminum cookpots are presented in Table 2. The median lead concentrations ranged from below the LOD to over 14,000 ppm (Pot #10). The highest lead concentrations were found in pressure cookers, where pressure relief vent pipes contained >10,000 ppm lead (Pot #9, 29, and 45–49). A typical lid of a traditional Afghan pressure cooker, depicting the vent pipes, is shown in Fig. 3. The highest detected lead concentration was 68,926 ppm in a vent pipe (Pot #9). The copper concentration in this vent pipe, like those in several other Afghan pressure cookers, was over 50%, suggesting that these inserts are likely brass or another lead-containing copper-based alloy (data not shown).

When sampled 15-min after the acetic acid had been brought to a boil (i.e., t = 15 min), lead concentrations in leachate ranged from 0.00006 micrograms per milliliter (µg/mL) (Pot #7) to 1.59 µg/mL (Pot #10). Estimated daily lead doses from one serving in these cookpots ranged from 0.015 to 398 micrograms per day (µg/day). The doses from 23 cookpots exceeded the childhood IRL and 16 were equal to or greater than the adult IRL (i.e., for women of childbearing age). Note that the estimated lead dose in all cookpots donated by the Afghan community exceeded the childhood IRL.

Additional leaching occurred in all cookpots after 24 h at room temperature (i.e., *t* = 24 h), by as much as 150-fold (Pot #29). Lead concentrations in leachate ranged from 0.0002 µg/mL (Pot #34) to 7.77 µg/mL (Pot #48). Estimated daily lead doses in these cookpots ranged from 0.050 to 1940  µg/day. The doses from 27 cookpots exceeded the childhood IRL and 23 exceeded the adult IRL.

### Stainless steel cookpots

We also conducted XRF and leachate analyses on five stainless steel items, including four pressure cookers and one stock pot (see Table 3).

The median lead concentrations ranged from below the LOD to 67 ppm (Pot #21). The two highest lead concentrations (104 and 132 ppm in Pots #21 and #28, respectively) were found in the pressure cooker vent pipes.

At *t* = 15 min, lead leachate levels ranged from 0.00018 to 0.0015 µg/mL, corresponding to estimated daily doses of 0.045 and 0.375 µg/day, respectively. At *t* = 24 h, lead leachate levels ranged from 0.00006 to 0.00054 µg/mL, corresponding to estimated daily doses of 0.015–0.135 µg/day. No stainless steel cookpots leached sufficient lead to exceed childhood or adult IRLs.

## Discussion

The results of this study suggest that aluminum cookpots brought into the U.S. from Afghanistan contain high lead levels. A local Afghan community member informed us that while traditional pressure cookers originate primarily from Afghanistan and Pakistan, inexpensive aluminum cookpots may be exported to Afghanistan from India, Pakistan, and China [53]. Foodstuffs prepared by Afghan families in traditional pressure cookers include meats, beans, chickpeas, potatoes, rice, tomatoes (fresh or paste), and a variety of spices [53]. The resulting dishes may be relatively acidic, thereby promoting the migration of lead and other metals from cookware.

It is noteworthy that, as of November 2021, traditional Afghan pressure cookers were available for purchase in the U.S. from online marketplaces (including eBay, AliExpress, Amazon, and Etsy). Several YouTube videos depict the use of Afghan pressure cookers by the non-Afghan population in the U.S. (https://www.youtube.com/results?search_query=afghan+pressure+cooker). In addition, some aluminum calderos, cookpots, steamers, and skillets purchased in the U.S. leached sufficient lead to exceed IRLs. Countries of origin included China, India, Colombia, and Taiwan.

While previous studies have demonstrated lead exposure from artisanal aluminum cookpots, the present study reports significantly higher concentrations. We found lead concentrations in components of Afghan pressure cookers in excess of 60,000 ppm and lead leachate levels that exceeded the FDA’s IRL for children by over 550-fold.

Our analysis of stainless steel cookpots confirmed previous findings of low lead levels [31], although relatively high levels of nickel and chromium have been observed in solutions and food substances with low pH [54–62]. This present study confirmed that some stainless steel cookpots release nickel and chromium. However, only one stainless steel pressure cooker (Pot #21) released relatively high levels of nickel (21.6 µg/mL) and chromium (72.2 µg/mL) after 24 h. These nickel and chromium concentrations were 17-fold and 126-fold higher, respectively, than the second highest observed concentrations. However, these metals are unlikely to pose a significant health concern for most individuals, other than those who exhibit allergic sensitization [62, 63]. Although none of the stainless cookpots leached sufficient lead to exceed IRLs, the presence of lead in safety valve vent pipes suggests that it is important to critically evaluate ancillary components of cookpots that may be made of brass or other lead-containing copper alloys [31, 42].

### Strengths and limitations of the study

A major strength of this study was our engagement with local Afghans, who provided cookpots used by their community. The XRF analyzer proved to be effective at screening cookpots, and our leachate method accounted both for cooking and storage of food in these containers, which we learned is a common practice in the community. The leachate analysis also provided an empirical measure of lead exposure, whereas the XRF data do not account for the presence of coatings (e.g., anodized finishes) that may impede migration of lead from cookware. We attempted to use commercially-available colorimetric tests to detect lead in some cookware, but it was necessary to abrade the cookpots’ surfaces to elicit a response, which was obscured by the presence of the abraded material.

Limitations include selection bias for some aluminum cookpots evaluated in this study, insofar as those donated by the Afghan community (15 of 40 tested) had previously been determined to contain high lead levels during in-home investigations. Because we do not have the necessary data to track the impact of removing these cookpots on children’s BLLs, we were not able to ascertain the relative contribution of this source to Afghan children’s overall lead exposure. We recognize that these children may have suffered high lead exposures in Afghanistan, which could contribute to their elevated BLLs. In addition, the in-home investigations identified other lead-containing items, including glazed dishes, silverware, spices, cosmetic jewelry, and personal care products (i.e., surma and kajal) [64]. Our ability to determine the effectiveness of removing sources of lead exposure is also compromised by the lack of follow-up BLL testing by some health care providers [64]. Because of safety concerns, we did not allow the pressure cookers to pressurize, and we allowed the acetic acid to boil for only 15 min. Therefore, the lead levels in leachate may be lower than what would be achieved under typical cooking practices. The relatively small number of stainless steel items evaluated limits our ability to better describe migration of metals from stainless steel. Finally, the assumption that both children and women of childbearing age consume 250 mL (i.e., 1 cup) per day from this type of cookware may under- or over-estimate their exposure.

## Conclusions and recommendations

To our knowledge, this is the first study to demonstrate that cookware brought to the U.S. by Afghan refugee families could represent an important source of lead exposure for both children and adults.

We are also not aware of any previous studies that have identified lead contamination of cookware available for purchase in the U.S. This poses a risk to all U.S. residents. We conclude that stainless steel is likely a safer alternative to aluminum, although it is important to determine whether any ancillary components are manufactured from lead-containing alloys, like brass.

Geopolitical events in Afghanistan have precipitated a humanitarian crisis, prompted most recently by the seizure of power by the Taliban in 2021. As a result, the U.S. is expected to resettle up to 95,000 Afghans [65]. Between 2010 and 2020, Washington State resettled 4166 Afghans holding Special Immigrant Visas (SIVs); 605 Afghan refugees arrived between October 1, 2020, and August 31, 2021 [66]. This influx of refugees combined with a national public health priority to reduce lead exposure should spur public health interventions at all levels.

Afghan refugees are not the only communities at risk from lead poisoning from cookware. Several studies have documented the manufacture of artisanal aluminum cookware from lead-containing scrap throughout the world. Our finding of high lead levels in cookpots imported from China, Taiwan, India, and Colombia confirmed that this is a worldwide public health problem. For example, we have recently found that other immigrant communities in King County may also be at risk; XRF analysis of an aluminum Idli maker (traditional to South India) revealed lead levels up to 1500 ppm (unpublished data). Future work will include testing cookware used by additional immigrant communities and ethnic restaurants in King County. In addition, based on the data generated in this study, we will further evaluate other potentially toxic metals (i.e., cadmium, cobalt, and manganese) that leach from these alloys.

### Recommendations

This study’s findings highlight the need to prevent the use of lead-containing scrap metal to manufacture cookware. As proposed by Weidenhamer et al. [35], the metal content of aluminum pots and other cookware produced around the world should be regulated, and a third-party certification should be developed that reflects a health-based standard. It is noteworthy that the World Health Organization (WHO) did not address artisanal aluminum cookware as a major source of lead exposure in its report on childhood lead poisoning [4]. Consequently, we recommend that WHO update its guidance to address this worldwide source of lead poisoning and institute international programs to address this issue.

The lack of a regulatory standard for lead content in metal cookware is a barrier to effective public health intervention. Consequently, we recommend that the FDA or other regulatory entities develop a standard method for lead analysis and a numerical standard for lead content. This standard could be adopted by agencies to inform regulations and health-based criteria.

We also recommend that the FDA align its IRL for children with the new CDC BLRV of 3.5 µg/dL. The FDA’s IRL of 3 µg/day is designed to maintain a child’s BLL below CDC’s old BLRV of 5 µg/dL [48]. Assuming a linear relationship between daily lead dose and associated BLL, the childhood IRL should be lowered to 2.1 µg/day.

We recommend that the FDA use its regulatory authority to prevent the importation and sale of lead-containing cookware in the U.S.

Efforts should be devoted to providing information about lead in cookware to immigrant communities. Sharing food is integral to Afghan culture and their pressure cookers are often regarded as family heirlooms. Therefore, information about this issue should also be included in the initial health screenings provided by public health programs upon entry to the U.S. It is also vital to engage community partners to deliver culturally competent messages that address behavioral barriers. Considering the broader public health implications of our findings, information campaigns should be developed to inform all U.S. residents of the hazards associated with some aluminum cookware.

In Washington State, only 4.4% of children 72 months of age and younger had BLL tests in 2016, compared to the corresponding national testing rate of 17.0% [67]. This relatively low testing rate in Washington State is a significant impediment to lead poisoning prevention efforts. Consequently, we support the recommendations provided in the King County Medical Society’s resolution to the 2018 annual meeting of the Washington State Medical Association to identify, treat, and eliminate sources of childhood lead poisoning in Washington State [68]. In particular, we support their recommendation that health care providers fulfill the federal obligation to conduct BLL testing of all Medicaid-eligible children at 12 and 24 months of age. It is also vital that all children previously determined to have elevated BLLs undergo follow-up testing by their health care providers.

Finally, Childhood Lead Poisoning Prevention Programs and local health jurisdictions lack adequate funding and cannot effectively track BLLs and intervene. Therefore, we recommend that adequate resources be provided by federal agencies to support lead surveillance, intervention, and data management. With the increasing number of refugee children being resettled in the U.S., additional resources are needed to meet CDC guidelines. Without these investments in public health infrastructure, our ability to prevent lead poisoning in our most vulnerable members of society will continue to be compromised.

## Supplementary Information

Supplemental Information

Supplementary table

Supplementary figure

## Data Availability

Data are available from the corresponding author upon reasonable request.
