# Supplementary material for: Investigating aluminum cookpots as a source of lead exposure in Afghan refugee children resettled in the United States
Source: J Expo Sci Environ Epidemiol. 2022 May 2;32(3):451–60. doi: 10.1038/s41370-022-00431-y (PMC9119854; doi:10.1038/s41370-022-00431-y)
Supplement: Supplementary file 2 — Supplementary table [file 41370_2022_431_MOESM2_ESM.docx]

| **Standard** | | | **XRF measurement** | | |
| --- | --- | --- | --- | --- | --- |
| **Name** | **Concentration (ppm)** | **Mean (ppm)** | | **Median (ppm)** | **Standard Deviation** |
| ERM-EB312a | 49.7 | 52.2 | | 52 | 3.63 |
| BAM EB316 | 87 | 65.4 | | 66 | 4.04 |
| AL 312/01 | 270 | 289 | | 288 | 15.1 |
| ERM-EB315a | 770 | 446 | | 454 | 11.5 |
| 511X G6026 | 2,770 | 3,173 | | 3,179 | 46.1 |
| 511X G6262B | 4,990 | 6,120 | | 6,130 | 57.0 |
| 511X G6012 | 11,000 | 10,800 | | 11,000 | 447 |
