# Supplementary material for: Investigating aluminum cookpots as a source of lead exposure in Afghan refugee children resettled in the United States
Source: J Expo Sci Environ Epidemiol. 2022 May 2;32(3):451–60. doi: 10.1038/s41370-022-00431-y (PMC9119854; doi:10.1038/s41370-022-00431-y)
Supplement: Supplementary file 3 [file 41370_2022_431_MOESM3_ESM.docx]

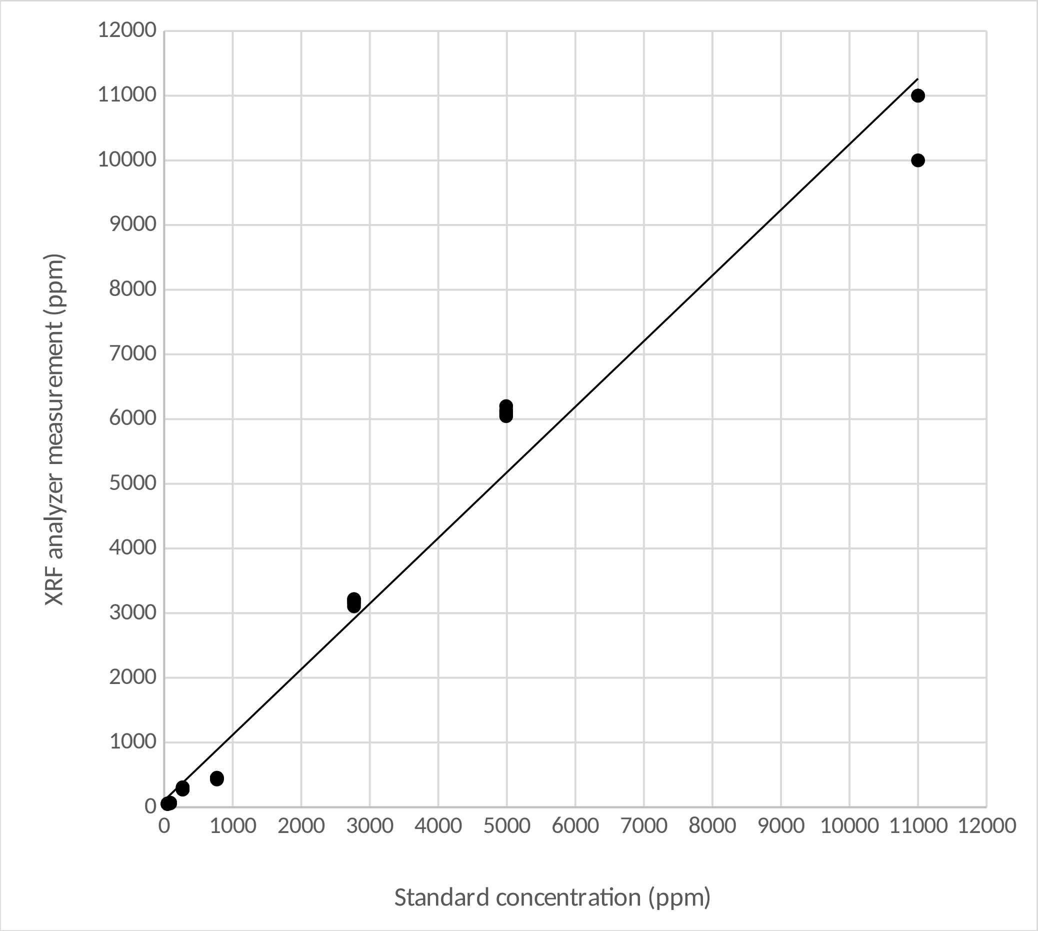

XRF analyzer measurement (ppm)
12000
11000
10000
9000
8000
7000
6000
5000
4000
3000
2000
1000
0
0 1000 2000 3000 4000 5000 6000 7000 8000 9000 10000 11000 12000
Standard concentration (ppm)
